# The Overlooked Burden of Food Insecurity among Asian Americans: Results from the California Health Interview Survey

**DOI:** 10.3390/ijerph15081684

**Published:** 2018-08-07

**Authors:** Monideepa B. Becerra, Salome Kapella Mshigeni, Benjamin J. Becerra

**Affiliations:** 1 Department of Health Science and Human Ecology, California State University, 5500 University Parkway, San Bernardino, CA 92407, USA; salome.mshigeni@csusb.edu; 2 School of Allied Health Professions, Loma Linda University, 24951 North Circle Drive, Loma Linda, CA 92350, USA; bbecerra@llu.edu

**Keywords:** Asian Americans, California Health Interview Survey, food security, Supplemental Nutrition Assistance Program (SNAP), acculturation, English language use

## Abstract

*Objective*: Food insecurity remains a major public health issue in the United States, though lack of research among Asian Americans continue to underreport the issue. The purpose of this study was to evaluate the prevalence and burden of food insecurity among disaggregated Asian American populations. *Methods*: The California Health Interview Survey, the largest state health survey, was used to assess the prevalence of food insecurity among Asian American subgroups with primary exposure variable of interest being acculturation. Survey-weighted descriptive, bivariate, and multivariable robust Poisson regression analyses, were conducted and alpha less than 0.05 was used to denote significance. *Results*: The highest prevalence of food insecurity was found among Vietnamese (16.42%) and the lowest prevalence was among Japanese (2.28%). A significant relationship was noted between prevalence of food insecurity and low acculturation for Chinese, Korean, and Vietnamese subgroups. Language spoken at home was significant associated with food insecurity. For example, among Chinese, being food insecure was associated with being bilingual (prevalence ratio [PR] = 2.51) or speaking a non-English language at home (PR = 7.24), while among South Asians, it was associated with speaking a non-English language at home was also related to higher prevalence (PR = 3.62), as compared to English speakers only. Likewise, being foreign-born also related to being food insecure among Chinese (PR = 2.31), Filipino (PR = 1.75), South Asian (PR = 3.35), Japanese (PR = 2.11), and Vietnamese (PR = 3.70) subgroups, when compared to their US-born counterparts. *Conclusion*: There is an imperative need to address food insecurity burden among Asian Americans, especially those who have low acculturation.

## 1. Introduction

The Asian American population is one of the fastest growing minority groups in the United States [1], yet, little research on health disparities exists for the group. One potential reason has been attributed to the model minority myth, which assumes Asians have unparalleled achievements in education and success [2], thus leading to the assumption that the population suffers little health disparities. Yet, studies demonstrate that such a myth has led to internalized racialism, further resulting in negative attitudes towards seeking mental health care and increased psychological distress [3].

Furthermore, Asian American data has been historically aggregated to present a homogeneous representation, resulting in the masking of more vulnerable subpopulations. Recent policy implementations, such as the White House Initiative for Asian Americans and Pacific Islanders [4], and the body of literature, demonstrates the importance of addressing the heterogeneity in the population. For example, Sakamoto, in evaluating the American Community Survey, demonstrated that when compared to whites, Asian Indians, Japanese, and Filipinos were less likely to be living in poverty, while Chinese, Koreans, Vietnamese, and several other Asian American subgroups were more likely to be in poverty [5]; hence contradicting the model minority myth. In an evaluation of hemorrhagic stroke risk among Asian Americans and other ethnic groups, Klatsky et al. [6] noted that while Asian Americans reported a higher risk of such stroke compared to whites, the rate was only explained by Japanese and Filipinos; thus demonstrating the heterogeneity in chronic disease risk among the Asian American population. Similarly, heterogeneity among Asian Americans has been noted in regards to health behaviors and chronic illnesses [7,8,9]. For example, results from a study addressing physical activity among Asian American subgroups utilizing CHIS data showed Chinese and Vietnamese subgroups who were bilingual were more likely to meet American College of Sport Medicine recommendations of physical activity level, as compared to those who reported only speaking a non-English language at home [10]. Undoubtedly, disaggregated research in the Asian American population is key to ensuring healthier outcomes of the nation’s population, as set forth by Healthy People 2020.

In recent years, food insecurity has gained national attention. Food insecurity, defined by the U.S. Department of Agriculture (USDA) as consistent access to and availability of enough food for all members of a household to lead an active and healthy lifestyle. The USDA further defines reduced quality, variety, or desirability of diet as low food security, which was historically called food insecurity without hunger, while the same characteristics with disrupted eating patterns reduction in food intake is considered very low food security, or historically known as food insecurity with hunger [11]. In 2016, 12.3% of U.S. households (42.2 million Americans), were reported to be food insecure. Furthermore, rates of food insecurity were found to be more prevalent among Hispanic and non-Hispanic Black households and those residing below the 185% poverty threshold [12]. Food insecurity has also been associated with negative health outcomes, including poor cognitive development [13,14], poor dietary choices [15,16], and mental illness [17,18]. For example, Weigel and group found higher rate of mental illness (including depression) among food insecure migrant and seasonal farmworkers [19]. Likewise, food insecurity with hunger was found to be substantially related to serious psychological distress among African-Americans [20], while low household food insecurity has been associated with adherence to physical activity guidelines among both children and adults [21]. Despite such empirical evidence, no current research exists on the burden of food insecurity among Asian Americans. As such, in this study we aimed to address this gap in the literature, by evaluating the period prevalence of food insecurity among disaggregation Asian American population using the largest state health survey.

Furthermore, we emphasized the role of acculturation in food insecurity among the population. The literature has identified acculturation, the process by which immigrants adapt to the host nation, as a major determinant of health disparities. For example, Tsunoda et al. [22] demonstrated that while Japanese adults in Japan perceived the time spent with children as appropriate for also drinking alcohol, Japanese Americans in Hawaii and California, on the other hand, perceived such a situation to be inappropriate. Ma and colleagues [23] further noted that cigarette smoking in homes was positively associated with being a new immigrant while less with increasing acculturation to the United States. Likewise, being more acculturated has been associated with higher fast food consumption among South Asian population in California [24]. While studies on the role of acculturation and food insecurity does not exist among Asian Americans, studies among other ethnic groups highlight putative relationship. For example, a study noted among West African refugees [25] noted that low acculturation was substantially related to higher rates of food insecurity, with similar trend noted among Puerto Ricans as well [26]. Despite such empirical evidence, studies on food insecurity and its potential relationship to acculturation is lacking. In fact, a recent study evaluating the burden of food insecurity, excluded Asian Americans from the study due to low sample [27]; thus further limiting the body of literature on the burden of food insecurity among the population. As such, our study addresses this critical gap in the literature. We hypothesize that the prevalence of food insecurity will be substantially different across the Asian American subgroups and less acculturated groups will likely have higher rates, putatively due to their limited knowledge or accessibility to food aid services.

## 2. Methods

### 2.1. Data Source

The public-use files of California Health Interview Survey (CHIS) adult section (2001, 2003, 2005, 2007, 2009, and 2011/2012) were used in this study. The study population was limited to Asian American subgroups: Chinese, Filipino, South Asian, Japanese, Korean, and Vietnamese.

### 2.2. Measures

The primary dependent variable was CHIS-provided variable on food insecurity, categorized in this study as food insecure versus food secure. CHIS provided a combined poverty and food insecurity variable as: at or above 200% federal poverty level (FPL), below 200% FPL and food secure, below 200% FPL and food insecure without hunger, below 200% FPL and food insecure with hunger. CHIS does not ask those at 200% or above their food security status. In this study, to ensure consistency with USDA guidelines, we refer to food insecure without hunger as low food security and food insecure with hunger as very low food secure. To assess food security level, CHIS researchers asked respondents the following questions: [1] “The food that (I/we) bought just didn’t last, and (I/we) didn’t have money to get more” [2] “(I/We) couldn’t afford to eat balanced meals,” [3] “In the last 12 months, did you or other adults in your household ever cut the size of your meals or skip meals because there wasn’t enough money for food?” [4] “How often did this happen—almost every month, some months but not every month, or only in 1 or 2 months?” [5] “In the last 12 months, did you ever eat less than you felt you should because there wasn’t enough money to buy food?” and [6] “In the last 12 months, were you ever hungry but didn’t eat because you couldn’t afford enough food?”, with the last variable assessing hunger. In this study, to ensure adequate sample size, we collapsed low food security and very low food security variables and refer to them as food insecure.

Primary independent variables included acculturation proxies of language spoken at home (Non-English only, English and another, English only) and country of birth (foreign-born vs. U.S.-born). Such measures have shown validity in the literature as proxies of acculturation and thus makes our results comparable to the empirical body of evidence on acculturation among Asian Americans. For example, Van Wieren and others [28] used CHIS data to explore acculturation and cardiovascular behaviors among the Latino population in California, and acculturation was assessed by country of birth. Likewise, An et al. [29] also utilized CHIS to assess how acculturation was related to cigarette smoking behaviors among Asian Americans where acculturation was assessed using language spoken at home.

Control variables included in regression analyses were: age (18–44 years, 45 years or more), sex (male or female), marital status (currently married or not currently married), education (high school or less, some college, bachelor’s degree or higher), employment status (currently employed or not currently employed), self-reported general health status (fair or poor vs. excellent, very good, or good), and zip code-based urban or rural residence, as such location may impact food insecurity due to availability of food items. Such variables were categorized based on CHIS-provided groups and/or natural breakpoints in the distribution. Additionally, body mass index (BMI) categories (overweight or obese, not overweight or obese) based on Asian BMI cutoffs [30] and survey year were included as controls. We chose to include BMI, though it is not a commonly utilized sociodemographic characteristics, as some studies have noted that BMI is related to food insecurity status among other populations [31,32]. Given that Supplemental Nutrition Assistant Program (SNAP) may alleviate food insecurity, we further assessed SNAP participation prevalence among the subgroups by citizenship status as a dichotomized variable.

### 2.3. Data Analysis

STATA v14 (StataCorp; College Station, TX, USA) was used for all analyses. Appropriate CHIS-provided jackknife survey weights were applied using the “svy” command to compute standard errors and obtain weighted prevalence estimates based on California population control totals. Chi-square analyses utilizing survey design-based *F* values were used to determine if there were significant differences in food insecurity prevalence among aforementioned control variables for each Asian American subgroup, in addition to SNAP participation by such subgroup stratified by citizenship status due to residential requirements for such federal aid programs. Survey-weighted Poisson regression, which utilizes a robust estimator by default in STATA [33], was run to estimate the adjusted prevalence ratios, according to Petersen and Deddens [34], of food insecurity by each Asian American subgroup as well as differences in SNAP participation by such subgroups. Finally, we also compared the food insecurity rates to the overall CHIS population for the study years. An alpha less than 0.05 was set for all analyses. The study was approved by the Institutional Review Board of California State University (approval number: 13086).

## 3. Results

A total of 24,803 Asian Americans, representing an average annual population estimate of 18,975,978, were included in this study. As displayed in Table 1, the highest period prevalence of food insecurity was noted among the Vietnamese subgroup (16.42%) and lowest among the Japanese subgroup (2.28%). Prevalence of speaking only a foreign language at home (acculturation proxy) was also highest among the Vietnamese subgroups (52.36%) and lowest among the Japanese (4.95%). Similarly, highest percent of foreign-born individuals (acculturation proxy) was noted among Vietnamese households (88.59%), with the lowest rate for foreign-born individuals among Japanese households (27.02%). Additional population characteristics are further displayed in Table 1.

As shown in Table 2, a significant relationship was found between prevalence of food insecurity and acculturation proxies for Chinese, Korean, and Vietnamese subgroups. For example, prevalence of food insecurity was reported to be 13.72% among non-English speaking Chinese households, as compared to 1.04% among English-only speaking households. Likewise, prevalence of food insecurity was higher among foreign-born Chinese households than those born in the United States (8.90% vs. 3.00%). Among Koreans, prevalence of food insecurity was significantly higher among non-English speaking households than their English-speaking counter parts (9.55% vs. 2.41%), with a similar trend noted for Vietnamese subgroup as well (23.46% vs. 4.84%). Similarly, when compared to those born in the U.S., food insecurity was more prevalent among foreign-born Vietnamese households (18.03% vs. 3.93%). As further noted in Table 2, several other characteristics were associated with food insecurity; and thus all variables were included in the full survey weighted multivariable regression analyses.

As shown in Table 3 (data on prevalence ratio [PR] for control variables is not shown), both acculturation proxies were associated with food insecurity among Asian Americans, though the relationships varied between subgroups. For example, speaking a language other than English at home was associated with 7.24 times higher prevalence of being food insecure, as compared to speaking English only, among the Chinese subgroup. Similarly, speaking English and another language was associated with nearly three times higher prevalence of food insecurity compared to only speaking English in the same population. South Asians speaking a non-English language at home also had over three and a half times higher prevalence of food insecurity, compared to those who reported speaking English only at home. Furthermore, prevalence food insecurity was significantly associated with being foreign-born among Chinese (prevalence ratio [PR] = 2.31), Filipino (PR = 1.75), Japanese (PR = 2.11), South Asian (PR = 3.35), and Vietnamese (PR = 3.70) subgroups.

Table 4 further displays the SNAP participation rate by acculturation status among the six Asian American subgroups. 

As noted, such participation is substantially low in the population over all. The highest rate based on language spoken at home was noted among Vietnamese who spoke a non-English only (15.67%) and were foreign-born (14.33%). Even when looking at by citizenship status, the prevalence was substantially low for all with the higher rates noted among non-citizens, especially among Vietnamese. For most subgroups, the participation rate was less than n = 10, thus resulting in lack of data reporting to ensure privacy of CHIS participants.

## 4. Discussion

While evaluation of the burden of food insecurity among minority populations is prevalent in the empirical body of literature, little assessment exists among the Asian American population. We thus studied the period prevalence of food insecurity among disaggregated Asian American subgroups in California, as well as whether acculturation was a determining factor of such disparities. The results of our study highlight several key findings: (1) food insecurity among Asian American subgroups is diverse, with lowest prevalence noted among Japanese (2.28%) and highest among Vietnamese (16.42%), (2) low acculturation is predominantly associated with higher prevalence of food insecurity among most Asian American subgroups, and (3) SNAP participation among the population remains substantially low.

Such results have several implications. In a previous study based in Los Angeles, Furness et al. noted that Whites, African-Americans, and Latinos had a higher prevalence of food insecurity compared to Asian/Pacific Islanders [35]. One plausible difference from such results to what is highlighted in our study is the disaggregation of data. Asian Americans are a diverse population with unique cultural and linguistic characteristics. Thus, the aggregation into one homogenous group can often mask true disparities among subgroups. Furthermore, in our study the highest prevalence of food insecurity was noted among the Vietnamese subgroup (16.42%), which is substantially higher than the other Asian American subgroup population as well as the entire CHIS population (11.80%). As such, consistent with the literature evaluating health disparities among Asian American, our study also demonstrate that Asian Americans remain a diverse population [36] with unique needs and thus disaggregation of data when assessing such social determinants of health are critical for public health efforts.

In addition, we noted that two proxies of acculturation were related to food insecurity among specific Asian American subgroups. This is similar to other studies that have shown Asian Americans who are less acculturated to suffer worse disparities. For instance, Tang et al. [37] noted that less acculturation was associated higher tobacco use while Jang and group [38] noted that alienation from heritage culture was associated with worse physical and mental health among Asian Americans.

One putative explanation for our results could be that less acculturated populations are more likely to adhere to Asian-based traditional food items, which are often more difficult to access due to cost [39], thus making such households more food insecure; however comprehensive assessment of Asian traditional food cost as compared to American food remains limited in the literature. In addition, in our study, we further see a substantially low SNAP participation in each Asian American subgroup, even among citizens. This could be potentially explained by culture-based stigma. For example, a report including Korean-speaking adults noted that most participants would not turn to a food assistance program for help and often considered them as a last option, often due to limitations of culturally appropriate food items [40] and culturally-associated stigma as such opportunities are often considered “handouts” [40]. The lack of any empirical evidence understanding the barriers to food aid participation among the Asian American population and the limitation of the aforementioned report to Korean population only, further highlights the imperative need for further research on understanding the barriers to ensuring food security among the Asian American population.

Finally, given the negative burden of food insecurity on health and behavioral outcomes, as noted in the literature, [18,21], the higher rates of food insecurity among less acculturated Asian American subgroups further shown in our study, the cumulative evidence warrants targeted public health efforts among the most at-risk groups. However, limited studies exist on what such public health efforts should include.

Herein also lies the opportunity for collaborative effort between the healthcare system and the community to ensure more positive outcomes. For example, in a proof of concept assessment of the efficacy of community health workers to improve childhood health outcomes, Martin et al. demonstrated the positive influence of home visitations on asthma control, emergency care utilizations, and inhaler usage [41]. While similar assessment on the efficacy of home visitation techniques on food insecurity remains limited, Tough et al. noted that home visitations improved nutrition counselling attendance among at-risk mothers, including those with language barriers [42]. As such, public health efforts to pilot test the efficacy of community health workers among Asian American subgroups and to create home visitation programs in order to assess food availability and increase participation in food assistance programs may help alleviate the burden of food insecurity among the most vulnerable Asian American populations.

Additionally, a critical point of contact for most populations remain the healthcare system. Means to identify Asian American subgroups at risk of food insecurity at healthcare facilities remains imperative. For example, the American Academy of Pediatrics notes the importance of a screening tool utilized during practice to identify children living in food insecure households; such as the Household Food Security Scale or the in-office 2-item screener [43]. A similar strategy can be utilized when screening adults, especially one tailored to Asian-specific languages.

Finally, as noted by Roncarolo and Potvin [44], simply providing access to food banks or food aid program is analogous to treating diseases with drugs. Instead, there is undoubtedly a need to identify the most at-risk populations early to prevent food insecurity from occurring. As such, to preventing the onset of food insecurity, if it were to be truly treated as a symptom of “social disease” [44], then governmental-level interventions, including that of local initiatives, are needed to improve continued access to healthy food options. For example, while farmers’ markets continue to be considered a key component of improving access to food, often they lack culturally appropriate food items. In San Francisco, California, a collaborative effort among food stamp programs and public health and nonprofit organizations demonstrated feasibility of increased access to farmers’ markets, especially through payment systems [45]. Similar strategies that incorporate partnerships between Asian American-based organizations and local public health agencies may provide a scope of improved access to food among such at-risk groups.

The results of our study should be interpreted in the context of some limitations inherent to the study design. The study sample is limited to California and thus cannot be generalized outside of the state. Furthermore, the proxies of acculturation utilized in this study may not encompass all feasible operationalization of acculturation. For example, studies note that acculturation can be bidimensional or unidimensional and these domains are not captured by the proxies. The self-report and recall biases inherent to surveys may further posit as limitations to interpretation of results. Nevertheless, such limitations do not negate the diversity in food insecurity prevalence noted in the Asian American subgroups, especially the disproportionately high levels noted among the Vietnamese subgroup.

## 5. Conclusions

Our study results demonstrate heterogeneity in the burden of food insecurity among the most vulnerable Asian American subgroups, especially those who are less acculturated. There is a significant gap in the literature addressing barriers to food aid among such populations and thus our results not only highlight the need for more comprehensive assessment, but also outreach to increase food aid participation for the most at-risk groups. There are also several strengths to this study. The sampling design of CHIS and survey-weighted analyses allow for generalization to Asian Americans in California, thus increasing the external validity of this study. Furthermore, the results provide one of the first assessments of food insecurity among Asian American subgroups, especially since there remains limited data to assess South Asian health, with CHIS being one of the few to provide public access to such data. As such, this study’s results provide a valuable addition by providing the first comprehensive analyses of the burden of food insecurity among disaggregated Asian American populations.

## Figures and Tables

**Table 1:** Study population characteristics by Asian American subgroup.

	Chinese	Filipino	South Asian	Japanese	Korean	Vietnamese
**Food insecure**						
No	6859 (92.4)	3506 (91.74)	2443 (96.86)	2325 (97.72)	3887 (93.43)	3732 (83.58)
Yes	488 (7.60)	259 (8.26)	90 (3.14)	52 (2.28)	308 (6.57)	854 (16.42)
**Language spoken at home**						
Non-English only	3204 (45.93)	563 (13.39)	351 (14.38)	135 (4.95)	2235 (44.3)	2791 (52.36)
English and another	2788 (38.31)	2008 (53.81)	1792 (70.88)	596 (26.51)	1600 (45.1)	1611 (42.22)
English only	1355 (15.76)	1194 (32.8)	390 (14.74)	1646 (68.54)	359 (10.6)	184 (5.424)
**Country of birth**						
Foreign-born	5790 (78.05)	2945 (72.69)	2289 (86.78)	652 (27.02)	3838 (82.62)	4370 (88.59)
U.S.-born	1557 (21.95)	820 (27.31)	244 (13.22)	1725 (72.98)	357 (17.38)	216 (11.41)
**Age (years)**						
18–44	3245 (54.39)	1782 (56.45)	1696 (74.79)	653 (33.61)	1760 (57.35)	1881 (56.77)
45 or more	4102 (45.61)	1983 (43.55)	837 (25.21)	1724 (66.39)	2435 (42.65)	2705 (43.23)
**Sex**						
Male	3103 (45.41)	1484 (45.87)	1352 (57.24)	926 (40.95)	1568 (39.00)	2263 (49.48)
Female	4244 (54.59)	2281 (54.13)	1181 (42.76)	1451 (59.05)	2627 (61.00)	2323 (50.52)
**Marital status**						
Not currently married	2647 (38.23)	1492 (42.62)	655 (29.42)	1096 (39.68)	1402 (39.67)	1595 (40.93)
Currently married	4700 (61.77)	2273 (57.38)	1878 (70.58)	1281 (60.32)	2793 (60.33)	2991 (59.07)
**Education**						
High school or less	1980 (31.9)	718 (23.58)	274 (12.14)	488 (26.54)	1334 (30.76)	2478 (51.93)
Some college	1162 (15.07)	927 (25.72)	263 (10.55)	632 (25.17)	570 (13.87)	833 (19.89)
Bachelors or higher	4205 (53.03)	2120 (50.7)	1996 (77.31)	1257 (48.3)	2291 (55.37)	1275 (28.18)
**Employment status**						
Currently employed	4598 (62.74)	2643 (70.07)	1857 (73.87)	1259 (54.34)	2097 (58.93)	2337 (59.20)
Currently unemployed	2749 (37.26)	1122 (29.93)	676 (26.13)	1118 (45.66)	2098 (41.07)	2249 (40.80)
**Self-rated general health status**						
Fair or poor	1574 (20.03)	605 (15.68)	189 (5.548)	296 (11.91)	1250 (22.89)	2249 (40.43)
Excellent, very good, or good	5773 (79.97)	3160 (84.32)	2344 (94.45)	2081 (88.09)	2945 (77.11)	2337 (59.57)
**Asian-specific BMI category**						
Not overweight or obese	3814 (53.37)	1272 (32.62)	978 (40.95)	993 (41.11)	2116 (53.97)	2430 (60.1)
Overweight or obese	3533 (46.63)	2493 (67.38)	1555 (59.05)	1384 (58.89)	2079 (46.03)	2156 (39.9)
**Urban/rural status**						
Urban	7162 (97.57)	3541 (95.12)	2427 (96.4)	2220 (95.04)	4092 (97.37)	4539 (99.27)
Rural	182 (2.43)	224 (4.88)	106 (3.60)	157 (4.96)	95 (2.63)	34 (0.73)

**Table 2:** Association between prevalence of food insecurity and study population characteristics, by Asian American subgroups, results of chi-square analysis.

	Chinese	Filipino	South Asian
Language spoken at home	<0.0001	0.316	0.0722
English only	1.04 (0.56, 1.94)	6.55 (4.04, 10.45)	1.12 (0.46, 2.69)
English and another	2.97 (2.19, 4.01)	9.04 (7.20, 11.29)	3.24 (2.35, 4.45)
Non-English only	13.72 (11.26, 16.59)	9.30 (6.59, 12.96)	4.73 (2.37, 9.22)
Country of birth	0.001	0.422	0.2622
U.S.-born	3.00 (1.52, 5.83)	6.91 (3.97, 11.75)	2.20 (1.16, 4.13)
Foreign-born	8.90 (7.40, 10.65)	8.76 (7.26, 10.54)	3.29 (2.42, 4.45)
Age	0.0032	0.1556	0.0428
18–44 years	5.57 (3.87, 7.96)	7.29 (5.30, 9.95)	2.66 (1.90, 3.73)
45 years or more	10.02 (8.42, 11.89)	9.51 (7.80, 11.54)	4.57 (2.94, 7.04)
Sex	0.9977	0.1846	0.3004
Male	7.60 (5.50, 10.43)	9.40 (6.94, 12.61)	2.73 (1.79, 4.17)
Female	7.60 (6.24, 9.22)	7.29 (5.86, 9.04)	3.69 (2.53, 5.36)
Marital Status	0.4084	0.0272	0.0036
Currently married	8.03 (6.38, 10.04)	6.74 (5.18, 8.71)	2.35 (1.62, 3.37)
Not currently married	6.91 (5.24, 9.06)	10.31 (7.84, 13.44)	5.06 (3.37, 7.54)
Education	<0.0001	<0.0001	<0.0001
Bachelors or higher	2.69 (1.75, 4.11)	4.25 (3.15, 5.71)	1.42 (0.89, 2.25)
High school or less	16.69 (13.57, 20.34)	17.19 (12.80, 22.69)	10.25 (6.28, 16.29)
Some college	5.67 (4.15, 7.70)	7.97 (5.76, 10.91)	7.62 (4.59, 12.39)
Employment status	0.0005	0.0001	0.0899
Currently employed	5.59 (4.08, 7.62)	6.15 (4.83, 7.81)	2.68 (1.89, 3.78)
Currently unemployed	10.98 (8.85, 13.54)	13.19 (9.90, 17.36)	4.46 (2.75, 7.15)
General health status	<0.0001	<0.0001	<0.0001
Excellent, very good, good	5.49 (4.22, 7.12)	6.80 (5.30, 8.69)	2.57 (1.88, 3.50)
Fair or poor	16.01 (12.67, 20.02)	16.11 (12.05, 21.20)	12.93 (7.65, 21.00)
Asian-specific BMI category	0.7872	0.0666	0.1769
Not overweight/obese	7.77 (6.00, 10.00)	6.57 (5.04, 8.53)	2.49 (1.56, 3.93)
Overweight/obese	7.41 (5.86, 9.32)	9.07 (7.19, 11.39)	3.60 (2.58, 5.00)
Urban/rural status	0.0163	0.3464	0.0589
Urban	7.72 (6.46, 9.20)	8.14 (6.71, 9.84)	2.96 (2.21, 3.97)
Rural	3.15 (1.47, 6.60)	10.57 (6.16, 17.54)	7.96 (2.90, 20.04)
	**Japanese**	**Korean**	**Vietnamese**
**Language spoken at home**	0.358	0.0005	<0.0001
English only	2.06 (1.22, 3.47)	2.41 (1.08, 5.29)	4.84 (1.66, 13.28)
English and another	2.45 (1.31, 4.54)	4.63 (2.89, 7.35)	9.18 (6.85, 12.19)
Non-English only	4.46 (1.86, 10.29)	9.55 (7.73, 11.75)	23.46 (20.81, 26.33)
**Country of birth**	0.0863	0.0932	<0.0001
U.S.-born	1.87 (1.08, 3.24)	3.02 (1.04, 8.44)	3.93 (1.74, 8.60)
Foreign-born	3.38 (2.14, 5.32)	7.32 (5.96, 8.97)	18.03 (15.99, 20.26)
**Age**	0.2102	<0.0001	<0.0001
18–44 years	3.05 (1.68, 5.47)	4.02 (2.72, 5.89)	12.46 (9.96, 15.47)
45 years or more	1.89 (1.15, 3.10)	10.01 (7.92, 12.57)	21.63 (19.09, 24.39)
**Sex**	0.2766	0.0221	0.0019
Male	2.88 (1.59, 5.17)	4.86 (3.65, 6.45)	13.30 (10.91, 16.11)
Female	1.87 (1.12, 3.10)	7.66 (5.87, 9.94)	19.48 (16.72, 22.56)
**Marital Status**	0.26	0.0245	0.5668
Currently married	1.87 (1.03, 3.38)	5.32 (4.21, 6.71)	16.88 (14.61, 19.41)
Not currently married	2.91 (1.75, 4.78)	8.47 (6.07, 11.70)	15.76 (12.85, 19.17)
**Education**	0.0335	<0.0001	<0.0001
Bachelors or higher	1.20 (0.71, 2.02)	2.43 (1.73, 3.41)	6.74 (4.39, 10.20)
High school or less	3.73 (1.97, 6.96)	13.18 (10.17, 16.91)	23.06 (20.57, 25.75)
Some college	2.84 (1.39, 5.72)	8.43 (4.93, 14.03)	12.80 (8.48, 18.86)
**Employment status**	0.3548	<0.0001	<0.0001
Currently employed	1.91 (1.00, 3.60)	3.99 (2.92, 5.44)	11.65 (9.26, 14.55)
Currently unemployed	2.73 (1.73, 4.29)	10.27 (7.92, 13.21)	23.34 (20.51, 26.43)
**General health status**	0.2197	<0.0001	<0.0001
Excellent, very good, good	2.10 (1.35, 3.25)	3.93 (2.91, 5.29)	10.22 (8.06, 12.86)
Fair or poor	3.63 (1.65, 7.76)	15.47 (11.89, 19.87)	25.56 (22.50, 28.88)
**Asian-specific BMI category**	0.7825	0.5507	0.0316
Not overweight/obese	2.42 (1.54, 3.79)	6.21 (4.67, 8.22)	14.72 (12.22, 17.62)
Overweight/obese	2.19 (1.22, 3.89)	7.00 (5.26, 9.25)	18.98 (16.34, 21.92)
**Urban/rural status**	0.192	0.1162	0.2329
Urban	2.34 (1.57, 3.48)	6.67 (5.42, 8.18)	16.45 (14.54, 18.55)
Rural	1.08 (0.34, 3.38)	2.48 (0.66, 8.84)	6.68 (1.16, 30.43)

**Table 3:** Prevalence ratio of food insecurity by acculturation status, among Asian American subgroup, results of multivariable robust Poisson regression analysis.

	Language Spoken at Home ^a^ PR (95% CI)			Country of Birth ^b^ PR (95% CI)	
	English Only (Reference)	English and Another	Non-English only	U.S.-Born (Reference)	Foreign-Born
Chinese	Ref.	2.51 (1.28, 4.94) **	7.24 (3.68, 14.24) ***	Ref.	2.31(1.17, 4.54) *
Filipino	Ref.	1.55 (0.98, 2.47)	1.56 (0.95, 2.55)	Ref.	1.75 (1.06, 2.87) *
South Asian	Ref.	2.53 (0.97, 6.64)	3.62 (1.04, 12.66) *	Ref.	3.35 (1.36, 8.20) **
Japanese	Ref.	1.24 (0.51, 3.00)	1.82 (0.71, 4.70)	Ref.	2.11 (1.09, 4.09) *
Korean	Ref.	1.57 (0.58, 4.23)	2.06 (0.73, 5.78)	Ref.	1.81 (0.67, 4.90)
Vietnamese	Ref.	1.56 (0.56, 4.40)	2.76 (0.99, 7.66)	Ref.	3.70 (1.58, 8.66) **

^a^ Poisson regression model includes language spoken at home as the primary exposure variable and control variables of age, sex, martial status, education, employment, self-reported general health status, urban/rural, BMI, and survey year; ^b^ Poisson regression model includes country of birth as the primary exposure variable and control variables of age, sex, martial status, education, employment, self-reported general health status, urban/rural, BMI, and survey year; PR = prevalence ratio, CI = confidence interval, Ref. = reference category; * *p* < 0.05, ** *p* < 0.01, *** *p* < 0.001.

**Table 4:** Prevalence of SNAP participation among Asian American subgroups.

	Language Spoken at Home			Country of Birth		Citizenship Status	
	English only	English and Another	Non-English only	U.S.-Born	Foreign-Born	Citizen	Non-Citizen
Chinese	--	2.95	4.77	--	4.28	1.3397	3.4626
Filipino	--	2.88	1.75	--	2.64	0.5794	2.9532
South Asian	--	3.35	--	--	3.48	0.7457	1.0001
Japanese	--	--	--	--	--	--	--
Korean	--	1..85	3.16	--	2.94	1.2389	1.3562
Vietnamese	--	9.02	15.67	3.12	14.33	6.7969	17.4218

-- The percent is not reported due to sample size being *n* < 10.
